# Paper-Based Analytical Devices for Accurate Assessment of Transferrin Saturation in Diagnosed Clinical Samples from Ischemic Stroke Patients

**DOI:** 10.1021/acs.analchem.3c01982

**Published:** 2023-07-24

**Authors:** Silvia Dortez, Núria DeGregorio-Rocasolano, Mònica Millán, Teresa Gasull, Agustín G. Crevillen, Alberto Escarpa

**Affiliations:** † Department of Analytical Chemistry, Physical Chemistry and Chemical Engineering, University of Alcala, 28805 Alcala de Henares, Madrid, Spain; ‡ Cellular and Molecular Neurobiology Research Group, Department of Neurosciences, Germans Trias I Pujol Research Institute (IGTP), 08916 Badalona, Barcelona, Spain; § Department of Neurociences, Germans Trias I Pujol University Hospital, Universitat Autònoma de Barcelona, 08916 Badalona, Barcelona, Spain; ∥ Department of Analytical Sciences, Faculty of Sciences, Universidad Nacional de Educacion a Distancia (UNED), 28040 Madrid, Spain; ⊥ Chemical Research Institute “Andrés M. Del Río” (IQAR), University of Alcala, 28805 Alcala de Henares, Madrid, Spain

## Abstract

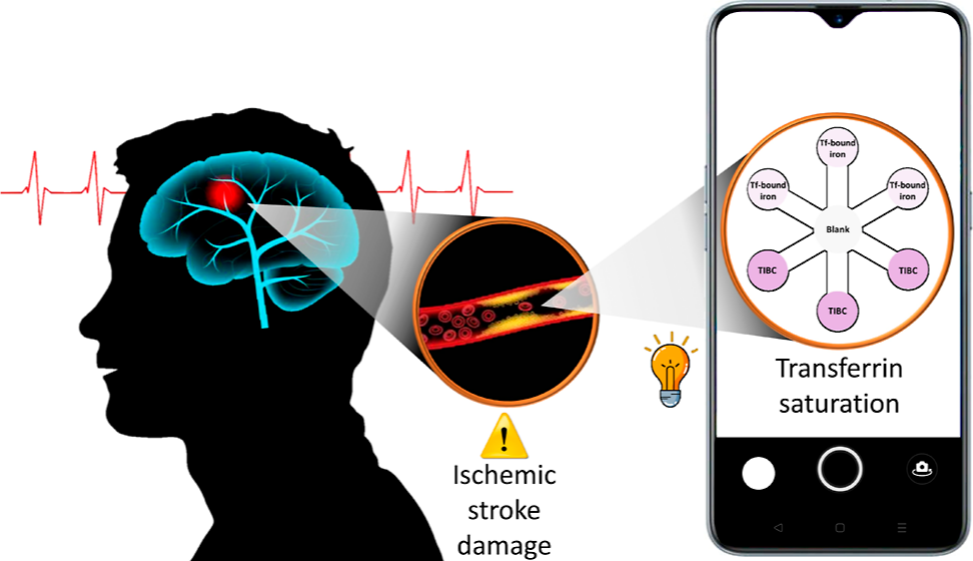

For the first time, a paper-based analytical device (PAD) was developed for the assessment of transferrin saturation (TSAT), which is defined as the ratio between iron bound to transferrin (Tf) and the total iron-binding capacity (TIBC) of Tf. Both parameters were simultaneously measured on the same PAD using ferrozine as a chromophore and a smartphone as the color reader. To this end, Tf was first isolated from serum using anti-Tf immunomagnetic beads to ensure that only the Tf-bound iron was measured, improving the selectivity and accuracy of TSAT assessment. To demonstrate the practical utility of the device, it was validated by analyzing a certified reference material, showing excellent accuracy (*E*_r_ < 4%) and good precision (RSD ≤ 6%). Finally, 18 diagnosed serum samples from ischemic stroke patients were analyzed by this approach, and the results were compared with those obtained by urea-PAGE, showing not only an excellent correlation (*r* = 0.93, *p* < 0.05) but that the PAD approach has become statistically identical to the free-interference urea-PAGE. In comparison with the slow, tedious, and non-miniaturized-PAGE, this PAD approach exhibited attractive characteristics such as low cost, disposability, and connectivity, showing great potential for future *point-of-care* testing, especially in developing countries and/or remote areas, where access to medical or clinical facilities is limited.

Transferrin (Tf) is a serum glycoprotein and the main iron transport source between body tissues. It can transport up to two ferric cations (Fe^3+^), existing three different Tf forms: apoTf (no Fe^3+^), monoferric-Tf, and diferric-Tf. The ratio between the amount of ferric iron bound to Tf and the maximum amount of iron that Tf can capture (total iron-binding capacity, TIBC) is defined as the percentage of transferrin saturation (TSAT). This clinical parameter reflects body iron states; therefore, it is used for diagnosing both iron deficiency and iron overload in combination with other serum biomarkers such as ferritin, serum soluble Tf receptor, and/or hepcidin.^1−4^ Moreover, a high level of TSAT has been proposed as a biomarker of the risk for various diseases, such as cardiovascular disease, diabetes mellitus, and cancer,^1,2^ and even several clinical and preclinical studies suggest that brain damage induced by ischemia is increased by a previous systemic iron overload.^5−7^

TSAT is typically calculated by the ratio between serum iron and TIBC (TSAT = [serum iron/TIBC] × 100). Both parameters are routinely measured by colorimetric approaches in clinical laboratories. For instance, serum iron is directly measured using ferrozine or bathophenanthroline chromophores.^1,8^ However, TIBC can be measured using direct or indirect methods. Regarding the direct methods, an additional stage is necessary concerning serum iron assessment, which is the addition of excess ferric iron to the serum sample to saturate all the free sites on Tf. Then, it is necessary to remove the excess unbound ferric iron by using an adsorbent agent,^1^ but some alternatives avoid this removal step.^8,9^ Furthermore, TIBC can be also calculated by measuring Tf concentration, considering that each mol of Tf can bind 2 mol of ferric iron.^10^ Regarding indirect methods, TIBC can be calculated by the sum of serum iron concentration and unsaturated iron-binding capacity (UIBC),^11^ which is defined as the additional amount of ferric iron needed to fully saturate Tf (TIBC = serum iron + UIBC).

However, these methods are not reliable in all clinical cases because they assume that all serum iron is bound to Tf,^12^ showing differences in the TSAT value up to 35% among some commercial methods.^13^ For example, they fail during intravenous or oral iron supplementation treatments^14,15^ because in this situation the level of non-Tf-bound iron is extremely high (up to 10 μM when TSAT > 70%).^16^ An interesting alternative is the direct determination of TSAT by urea-PAGE (it allows the separation and detection apoTf, monoferric-Tf, and diferric-Tf) because it has proven to be more accurate and reliable than the aforementioned methods.^15,17,18^ However, the PAGE technique is slow, tedious, and lacks automation and miniaturization, which hamper its use in decentralized scenarios.

In this sense, the development of new bioanalytical approaches that allow for the accurate determination of TSAT and with potential use and development of future *point-of-care* tests (POCTs) is highly needed. Indeed, low-cost POCTs are an interesting and relevant alternative for determining TSAT, especially to facilitate the diagnosis of iron deficiency anemia in developing countries^3^ and/or when a fast decision must be taken (such as the evaluation of the growth and extent of stroke damage).^18^ To the best of our knowledge, there is currently no POCT for TSAT assessment. POCT is a top priority for the World Health Organization, especially, in resource-limited settings.^19^ Among POCT technologies, the combination of smartphone-based and paper-based assays is attracting enormous attention from the scientific community because the smartphone provides straightforward colorimetric detection, portability, and connectivity, and paper provides extremely low-cost analytical platforms, which are characteristics highly demanded by developing countries.^20,21^

In the designing and developing of a novel POCT, paper-based analytical devices (PADs) offer several advantages such as inexpensive production, portability, operational simplicity, miniaturization, biocompatibility, minimal reagent consumption, disposability, and capillary flow.^22−24^ There are interesting examples of (μ)PADs with smartphone-based detection for serum iron^25−27^ and serum ferritin quantification (lateral flow assay)^28^ but, to our best knowledge, there is no article reporting on TSAT assessment using a PAD approach.

In this work, we propose a new approach to measure TSAT, which is based on a colorimetric PAD with smartphone reading using anti-transferrin immunomagnetic beads (anti-Tf-MBs). The approach was validated using a certified reference material (human serum), and then it was applied to the analysis of diagnosed serum samples from ischemic stroke patients. These results were compared with those obtained by a well-established free-interference method (urea-PAGE).

## Materials and Methods

### Reagents and Materials

Iron (III) chloride hexahydrate, ferrozine (3-(2-pyridyl)-5,6-diphenyl-1,2,4-triazine-*p*,*p*′-sulfonic acid monosodium salt hydrate), hydroxylamine hydrochloride, acetic acid glacial, and sodium acetate anhydrous were purchased from Merck (Darmstadt, Germany). Tris was purchased from Roche (Basel, Switzerland). Potassium chloride and disodium hydrogen phosphate were purchased from Scharlau (Barcelona, Spain). Sodium dihydrogen phosphate was purchased from PanReac (Barcelona, Spain). Pierce Protein G Magnetic Beads and sodium chloride were acquired from Thermo Fisher Scientific (USA). Human serum (certified reference material, Spintrol H normal 1002121) was purchased from Spinreact (Girona, Spain). Anti-Tf antibody (ab66952) was purchased from Abcam (Cambridge, UK).

Anonymized serum samples of the multicenter, randomized, double-blind, placebo-controlled TANDEM-1^7^ (Thrombolysis and Deferoxamine in Middle Cerebral Artery occlusion) study were used to evaluate TSAT by PAD and compared with the urea-PAGE method (as explained in DeGregorio-Rocasolano et al.^18^). The serum samples used were specifically those collected at the Hospital Universitari Germans Trias I Pujol (HUGTP) in untreated patients. All samples were stored at −20 °C until use.

The TANDEM-1 study was approved by the Spanish Drug Agency (eudraCT 2007-0006731-31) and by local Ethics Committees including the HUGTP Ethics Committee and was registered in clinicalTrials.gov as NCT00777140.

A stock solution of 2 M acetate buffer pH 4.8 was prepared by dissolving appropriate amounts of acetic acid and sodium acetate in Milli-Q water. A stock solution of 2 M Tris buffer pH 7.6 was prepared in Milli-Q water. A stock solution of 0.1 M phosphate buffer saline (PBS) pH 7.6 was prepared by dissolving appropriate amounts of sodium dihydrogen phosphate, disodium hydrogen phosphate, sodium chloride, and potassium chloride in Milli-Q water. The solution of hydroxylamine was prepared by dissolving appropriate amounts in 2 M acetate buffer of pH 4.8 to a concentration of 100 mg mL^–1^. The solution of ferrozine was prepared by dissolving appropriate amounts in 2 M acetate buffer of pH 4.8 to a concentration of 49 mg mL^–1^. Iron (III) standard solutions were daily prepared by dissolving appropriate amounts in 100 mg mL^–1^ hydroxylamine solution (prepared in 2 M acetate buffer of pH 4.8) or in 2 M Tris buffer of pH 7.6 to a concentration of 1 mg mL^–1^.

All reagents and solvents were of analytical grade. All solutions were prepared in Milli-Q water (Merck Millipore, Darmstadt, Germany).

Whatman Chromatography Paper 1 CHR was purchased from Merck (Darmstadt, Germany). An Invitrogen DynaMagTM-2 Magnet was purchased from Thermo Fisher Scientific (USA). The waterproof marker pen Lumocolor permanent CD/DVD/BD 310 was purchased from Staedtler (Nuremberg, Germany). Tesa 4024 clear packing tape and Samtian F40II Lightbox with LED were purchased from Amazon (Spain).

### Instrumentation

AutoCAD 2018 (Autodesk, Student Version) was used to design the PAD, and they were drawn on a sheet of filter paper (Whatman 1 CHR) using a desktop cutting plotter (Silhouette Cameo 3, Silhouette).

Photos were taken using a Realme X2 mobile and a lightbox (dimensions 44 × 44 × 44 cm, including 84 brightness LED light beads inside). Subsequent image analysis was performed with ImageJ software.

### Procedures

#### PAD Design and Fabrication

Whatman 1 CHR was used as the filter paper because it is hydrophilic, homogeneous, reproducible, biocompatible, and cheap, among others.^29^

To draw the pattern of the PAD, AutoCAD software was used. The pattern was transferred to a piece of filter paper using a cutter plot, in which the blade was replaced by a waterproof marker pen. Then, the waterproof marker pen ink penetrated the paper to form the hydrophobic walls on the filter paper. Once the PADs were drawn on the filter paper, they were individually cut using the cutter plot (the blade was reinstalled), and it was checked whether the hydrophobic walls were well-created with the waterproof marker pen on both sides of the device. Next, the backside of the printing surface was covered with clear packing tape to prevent the solution from leaking out underneath the PAD.^27^

The device consisted of six circular zones (detection reservoirs, 10 mm diameter) at the ends of six channels (10 mm length and 5 mm width), in a radial configuration where only the circular zones were used, assuring that all reservoirs are at the same distance from the point of light. This fact is very important since in this application, two different parameters (Tf-bound iron and TIBC) were measured simultaneously, which allows the accurate assessment of the TSAT as a ratio of both. Furthermore, there was another detection reservoir at the junction of the six channels. The selected device was selected for the described novel application considering that it is one of the most ubiquitous designs in the literature, the aspect that would facilitate other researchers to adopt the described methodology without any significant changes.

Ferrozine was used as a colorimetric agent because it is commonly used for the colorimetric iron assay.^30−32^ The optimal conditions of this chromogenic reagent were chosen based on our previous article.^27^ 0.5 μL of 49 mg mL^–1^ ferrozine in 2 M acetate buffer of pH 4.8 was added to each detection reservoir of PAD, and it was dried completely in an oven at 60 °C for 2 min. Then, the PAD was ready to use.

#### Immunopurification Step for Tf-Bound Iron Isolation

Anti-Tf-MBs were used for Tf isolation in serum samples and were prepared according to the literature.^33,34^ This step allowed us to isolate Tf-bound iron from the rest of the iron (III) species present in serum, ensuring that we only measured iron bound to Tf in the following steps.^27^

(i) Immunoreaction: 5 μL of anti-Tf-MBs was resuspended in 150 μL of serum sample (which contained Tf) and this vial was incubated at 25 °C and 950 rpm for 45 min (Eppendorf ThermoMixer C, Hamburg, Germany).
(ii) Cleaning: the vial was placed on the magnet holding block for 2 min, and then, the supernatant was removed. Two washing steps were carried out with 100 μL of 0.1 M PBS solution of pH 7.4.
(iii) Reduction of Fe^3+^ to Fe^2+^: Tf-anti-Tf-MBs were resuspended in 15 μL of 100 mg mL^–1^ hydroxylamine (reduction agent), prepared in 2 M acetate buffer of pH 4.8. This solution was incubated for 30 min in a thermoshaker at 25 °C and 950 rpm, releasing iron from Tf and reducing Fe^3+^ to Fe^2+^. Finally, the vial was placed on the magnet holding block for 2 min, and 5 μL of supernatant was transferred onto the reservoir of the PAD.

For TIBC assessment, besides previous steps, a new one (iron saturation) was carried out between (ii) and (iii) (see Figure S1):

(ii, b) Iron saturation: Tf retained by anti-Tf-MBs was saturated with iron (III) using 10 μL of 10 μg mL^–1^ iron (III) standard solution in 2 M Tris buffer pH 7.6 and incubated for 15 min, at 25 °C and 950 rpm. After that, the vial was placed on the magnet holding block for 2 min, and then, the supernatant was removed. Two washing steps were carried out with 100 μL of 0.1 M PBS solution of pH 7.4.

#### TSAT Colorimetric Assay Using PAD

The PAD contains seven detection reservoirs: three were used for Tf-bound iron measurement, the other three for TIBC assessment, and the last for the blank (100 mg mL^–1^ hydroxylamine in 2 M acetate buffer of pH 4.8).

The assay was as follows: 5 μL of each sample was added to the PAD and then, the paper turned purple after 10 min of reaction between Fe^2+^ and ferrozine. The reaction was carried out at room temperature. The intensity of the purple color was directly proportional to the iron concentration.

Images of the PAD were taken by a smartphone (Realme X2). The whole imaging process was performed inside a professional photography box, where the smartphone was on top. In addition, the PAD was placed 22 cm below the smartphone, keeping the same reservoir–smartphone camera distance.^27^

The images were analyzed by ImageJ software in the red–blue–green color format. The green channel was selected because it provides the maximum intensity due to it being the complementary color to the purple of the colored product of the reaction of ferrozine with iron.^27^ Next, the image was inverted (pure white and black backgrounds were considered to be of 0- and 255-pixel intensity, respectively), and the detection zones for each reservoir of the PAD were selected individually (circular diameter of 60 pixels) to calculate the mean intensity of the selected area, subtracting the blank value.

Finally, TSAT was calculated by dividing the concentration of Tf-bound iron by TIBC (μg mL^–1^).
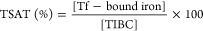
1

#### Calibration Curves

External calibration linear plots were constructed using Fe^3+^ standard solutions. 10 μL of the Fe^3+^ standard solution was mixed with 10 μL of 100 mg mL^–1^ hydroxylamine solution (in 2 M acetate buffer pH 4.8) in a vial. Then, it was kept under agitation for 15 min at 25 °C and 950 rpm using a thermoshaker. In this process, Fe^3+^ was reduced to Fe^2+^. Next, 5 μL the previous mixture was added onto the detection zone of the PAD, where the paper turned purple after 2 min of the reaction between Fe^2+^ and ferrozine (at room temperature). A blank (100 mg mL^–1^ hydroxylamine in 2 M acetate buffer of pH 4.8) was also prepared and analyzed in the same way. Finally, the images were taken and processed in the same way as in the previous section (TSAT colorimetric assay using PAD).

## Results and Discussion

### Analytical Design and Optimization of the PAD-Based Approach for TSAT Assessment

**Figure 1:**
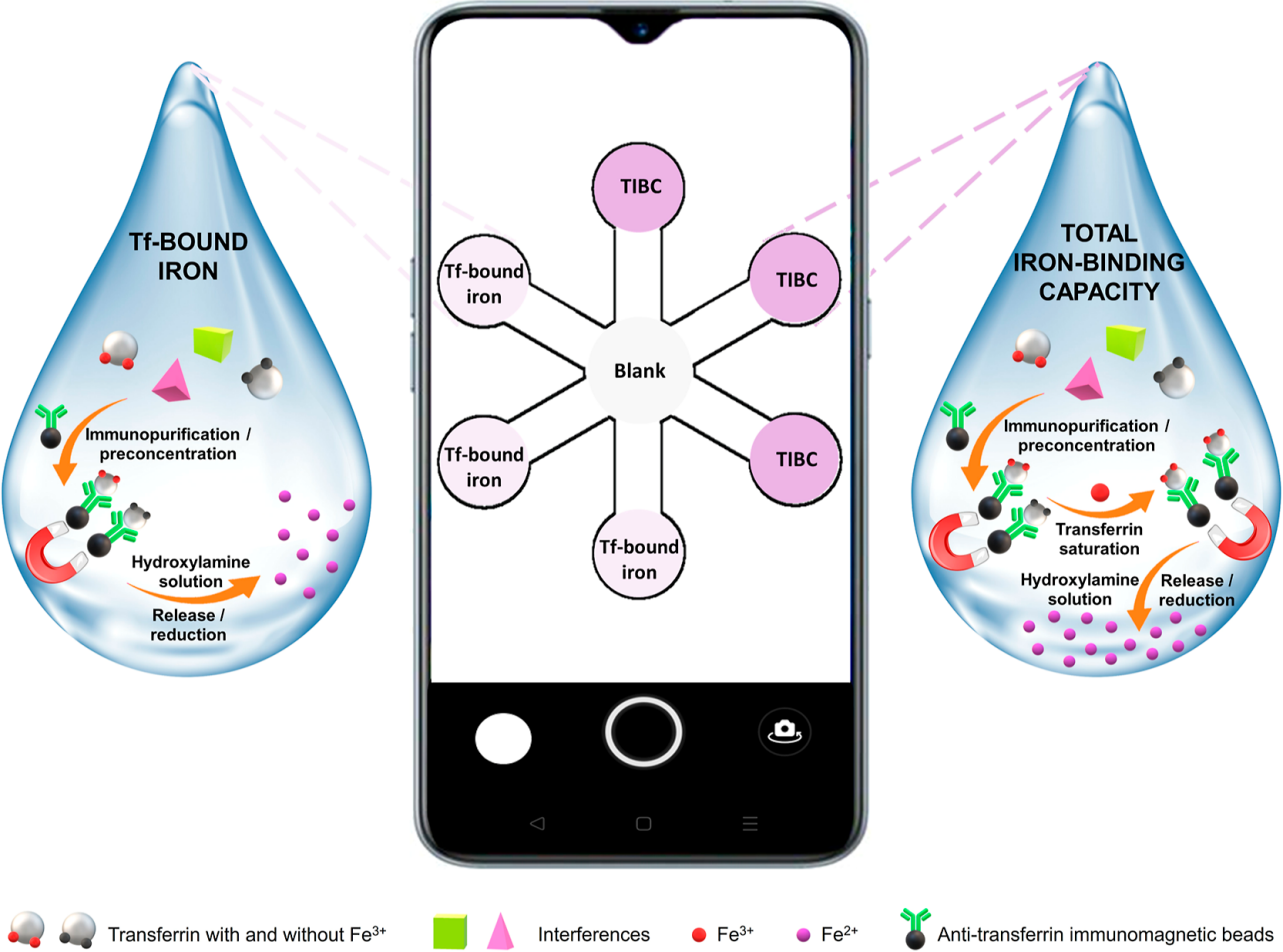
Strategy for the determination of TSAT using anti-Tf-MBs and the PAD-smartphone sensor.

Figure 1 shows the analytical strategy for the assessment of the clinical parameter TSAT. First, Tf was isolated from the serum sample using anti-Tf-MBs to ensure that only iron bound to Tf is measured. Then, the assessment of TSAT was carried out by a parallel and simultaneous colorimetric measurement on the same PAD of two parameters: (i) the Tf-bound iron was present physiologically in the sample and (ii) TIBC (after the saturation of Tf with Fe^3+^). The methodology for Tf-bound iron assessment was previously developed by our group,^27^ so we first develop a method for TIBC assessment, and then both methodologies were combined for designing a novel approach for TSAT assessment (see eq 1) using unique diagnosed serum samples from ischemic stroke patients.

TIBC is defined as the maximum amount of iron (III) that saturates all serum Tf. Our methodology for TIBC assessment consists of four steps: (i) Tf isolation from serum sample using anti-Tf-MBs; (ii) saturation of the isolated Tf with a Fe^3+^ solution; (iii) release of Fe^3+^ from isolated Tf and reduction to Fe^2+^; and (iv) determination of Fe^2+^ using the colorimetric PAD.

**Figure 2:**
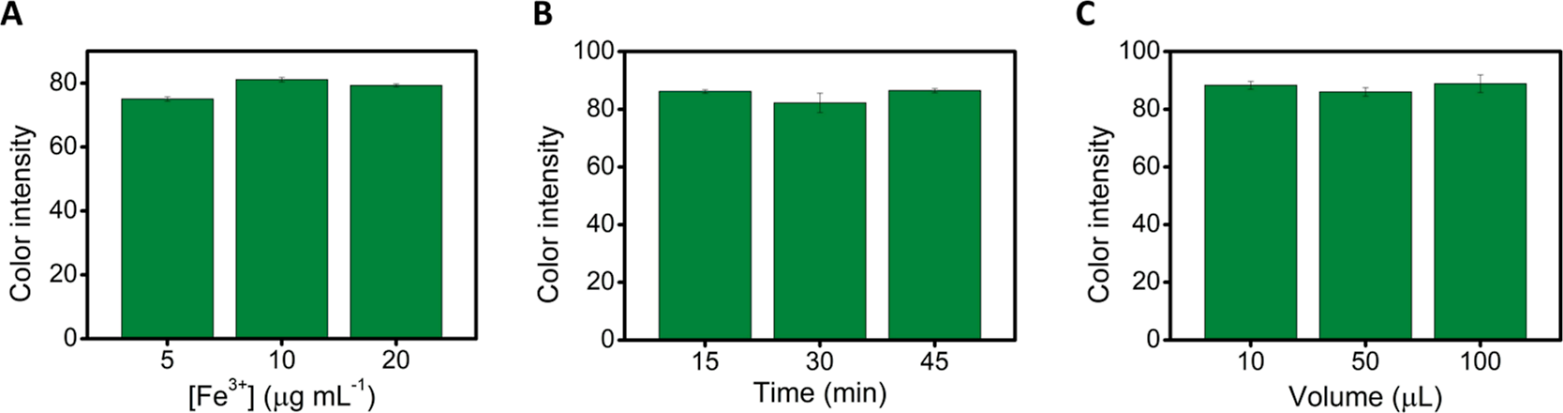
Color intensity (green channel) using the immunopurification step and the smartphone-PAD sensor: (A) At different concentrations of 10 μL of Fe^3+^ in 2 M Tris buffer of pH 7.6 during 15 min for Tf saturation. (B) At different Tf saturation times using 10 μL of 10 μg mL^–1^ Fe^3+^ in 2 M Tris buffer of pH 7.6. (C) At different volumes of 10 μg mL^–1^ Fe^3+^ in 2 M Tris buffer pH 7.6 solution during 15 min (*n* = 3). For other experimental conditions see the Immunopurification step for the Tf-bound iron isolation section.

The concentration of Fe^3+^ solution for Tf saturation (TIBC assessment) was first studied. Solutions of 5, 10, and 20 μg mL^–1^ Fe^3+^ in 2 M Tris buffer pH 7.6 solution were selected to saturate a solution of 2.03 g L^–1^ Tf (after immunopurification). The results showed that the highest color intensity was obtained using 10 μg mL^–1^ Fe^3+^, so it was chosen as the best iron concentration to saturate Tf for the rest of the experiments (see Figure 2A). The next study consisted of optimizing the reaction time to saturate Tf with 10 μg mL^–1^ Fe^3+^ in 2 M Tris buffer pH 7.6 solution. Reaction times of 15, 30, and 45 min were studied. No significant differences were obtained between the different times, so 15 min was chosen (see Figure 2B). Finally, the added volume of 10 μg mL^–1^ Fe^3+^ solution was also optimized. Volumes of 10, 50, and 100 μL were assayed. As for Tf saturation time, no significant differences were obtained between the different volumes. For this reason, 10 μL was selected as the optimum volume for Tf saturation (see Figure 2C).

### Analytical Performance

Then, the analytical performance of the PAD was carefully evaluated. Linear calibration plots were constructed by analyzing Fe^3+^ standard solutions from 5 to 40 μg mL^–1^ with three different PADs (see Figure S2). As Figure S2 inset shows, the intensity of the color formed depended on the iron concentration. A complete calibration plot was constructed using a single PAD. The calibration plot for iron was linear in the range of 5–40 μg mL^–1^, showing an excellent correlation coefficient (*r* = 0.997). The calibration slope was 0.77 ± 0.03 px mL μg ^–1^ and the intercept was 3.8 ± 0.6 px (*n* = 3). In addition, the reproducibility of calibration slopes using different PADs was very good (RSD = 3%).

However, the limit of detection (LOD) was 2 μg mL^–1^ (calculated as 3*s*_a_/*b*, where *s*_a_ is the standard deviation of the intercept), which is not enough to detect iron in human serum (normal range from 0.7 to 1.7 μg mL^–1^).^35^ However, this is not a problem when serum samples were analyzed because an immunopurification step was implemented for the isolation of Tf using anti-Tf-MBs,^33,34^ yielding a 10 times preconcentration (Tf present in 150 μL serum is concentrated in a 15 μL solution during the immunopurification). In this sense, the LOD of the overall method was reduced from 2 to 0.2 μg mL^–1^. Moreover, this step ensures that only Fe^3+^ bound to Tf is measured, avoiding the interference of non-Tf-bound iron and improving the accuracy of TSAT evaluation.

Table 1 lists the results obtained for the determination of Tf-bound iron and TIBC in a certified reference material (human serum) (*n* = 3). In addition, both parameters were measured on the same PAD, demonstrating its multiplexing capability and highlighting their practical use (see Figure S3). Based on these results, our method showed excellent accuracy and precision.

**Table 1:** Analysis of Certified Reference Material by PAD^a^

parameter	certified value (μg mL^–1^)	PAD value (μg mL^–1^)		*E*_r_ (%)
		*x̅* ± *s*	RSD (%)	
serum iron	1.09	1.05 ± 0.01	1	4
TIBC	2.85	2.8 ± 0.2	6	2

a Values are expressed as mean value ± SD (*n* = 3).

On studying these data thoroughly, the quantification values for serum iron and TIBC were excellent and reproducible, although always below the certified value. This might be because our method only measured the iron bound to Tf, while the reference values were obtained by measuring all Fe^3+^ in the serum (serum iron). However, we must bear in mind that this work aims to evaluate TSAT, so the Fe^3+^ not bound to Tf must be not considered. This event would improve the accuracy of the TSAT assessment. If TSAT is calculated for the certified reference material (using eq 1, these data are not provided in the certificate), the value is 38.2%, which is slightly higher than that given by our method (37.6%).

**Figure 3:**
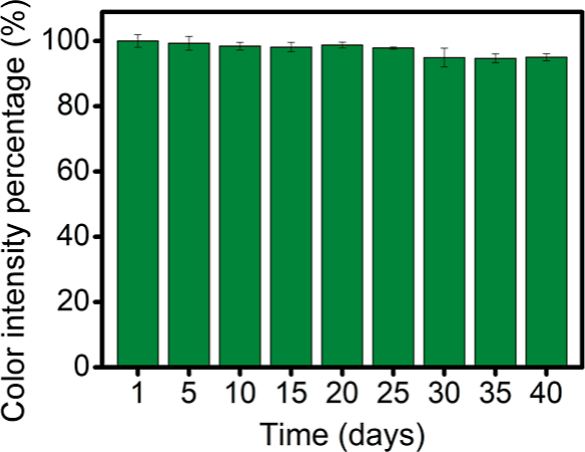
Stability of colorimetric PAD (in plastic zipper bags at room temperature and protected from light). Experimental conditions: 5 μL of 500 μg mL^–1^ Fe^3+^ standard solution prepared in 100 mg mL^–1^ hydroxylamine solution (2 M acetate buffer pH 4.8) and 2 min reaction time (*n* = 3).

The storage stability of the PADs was also evaluated for practical purposes. To this end, several PADs were prepared and stored in plastic zipper bags at room temperature and protected from light. The color intensity generated in these devices (three different PADs) was measured over time. Figure 3 represents the percentage variation of the color intensity over time concerning the color intensity obtained on the first day. After 40 days, there were no significant differences in color intensity compared with the first day (test *t*, *p* < 0.05, two sides, *n* = 3), so the PADs are stable for at least 40 days under the selected storage conditions.

### Assessment of TSAT in Serum Samples from an Ischemic Stroke Patient

**Figure 4:**
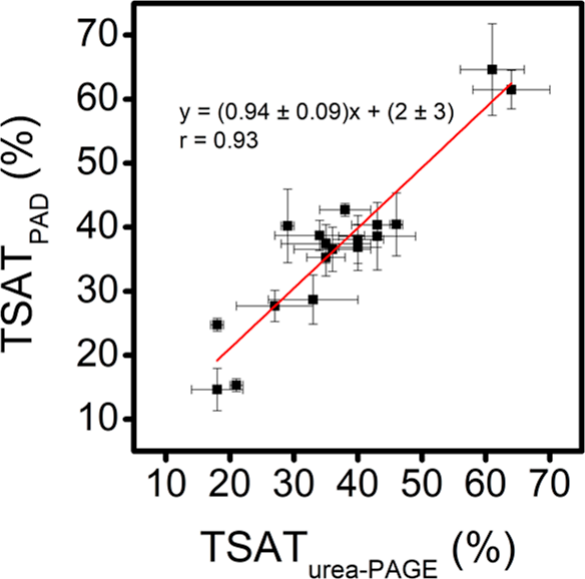
Correlation between TSAT values obtained by PAD and urea-PAGE (*n* = 3). Experimental condition: see the section Immunopurification Step for Tf-Bound Iron Isolation.

To demonstrate the applicability of the proposed PAD in clinical samples, TSAT was measured in 18 anonymized serum samples from ischemic stroke patients of the TANDEM-1 study. The samples were analyzed following our approach and the obtained TSAT values were compared with those obtained by the urea-PAGE method^18^ (see Table S1). In addition, Figure 4 shows the correlation plot between both sets of data, exhibiting a good correlation coefficient (*r* = 0.93). More importantly, both methods did not show significant differences at the 95% level since the values obtained for slope (0.94 ± 0.09) and the intercept (2 ± 3), included 1 and 0, respectively, revealing an excellent accuracy since the urea-PAGE method is a free-interference method.

On the other hand, the urea-PAGE method separates and detects the different forms of Tf (apoTf, monoferric-Tf, and diferric-Tf), so it measures the TSAT directly without considering the iron not bound to Tf, but it takes 18 h. Our approach takes less than 2 h and showed not only an excellent correlation but a high agreement in terms of accuracy with the free-interference urea-PAGE even at high TSAT (see Table S1, S17, and S18 samples), meaning that our strategy, based on the immunopurification of Tf and the colorimetric PAD, only measures the Tf-bound iron. This contrasts with results provided by other colorimetric methodologies that rely on the measurement of total iron serum (bound and not bound to Tf).

Moreover, current methods for TSAT assessment employ automatic benchtop equipment,^7,11^ which is expensive and located in clinical laboratories or medical centers. To our best knowledge, there is no PAD or POCT for TSAT assessment reported in the literature, so our PAD approach is the pioneer in this application and, interestingly, a potential candidate for a future POCT. Indeed, the use of a processed sample (serum) and the multistep procedure of our approach does not meet the user-friendly feature required in POCT devices yet;^24^ however, it meets other important features such as being affordable, sensitive, specific, and deliverable to end-users. Moreover, our PAD can be used by any skilled health professional and even blood samples may be analyzed instead of serum, thanks to the use of anti-Tf-MBs (not assayed).

## Conclusions

A new approach to accurately assess TSAT was successfully developed by measuring the Tf-bound iron and TIBC on a single PAD using a smartphone as a color reader. Thanks to its multiplexing capabilities, Tf-bound iron and TIBC assays were simultaneously measured on the same PAD, eliminating the inter-PAD variability and the variability due to changes in external factors such as light intensity, temperature, humidity, and smartphone model, among others, that can affect the analysis.

Importantly, the use of anti-Tf-MBs allowed us to measure only the Tf-bound iron, avoiding inaccuracies in TSAT evaluation, even at high values due to the presence of non-Tf-bound iron.

Our approach was validated by analyzing a certified reference material, showing excellent accuracy (recoveries > 96%) and good precision (RSD ≤ 6%). Moreover, it was successfully applied to the analysis of serum from ischemic stroke patients, showing a very good correlation and excellent accuracy with a reference method (urea-PAGE). Despite the analysis time (about 1:30 h.), it was a distinctive feature compared to the urea-PAGE, which takes 18 h.

Even more exciting are the opportunities our approach opens up. It has several interesting features such as low cost, disposability, and connectivity to be the potential basis of a future POCT for TSAT assessment, making it very attractive for use in developing countries or remote areas, where access to medical or clinical facilities is restricted.

## Supporting Information Available

Scheme of the analytical reactions used for determination of TSAT; image of a PAD used for the calibration curve (with the corresponding calibration plot) and multiplexing analysis; and table with TSAT values of serum samples from ischemic stroke patients obtained by a PAD and urea-PAGE (PDF)

## Abbreviations

Anti-Tf-MBs: anti-transferrin immunomagnetic beads
LOD: limit of detection
MBs: immunomagnetic beads
μPAD: microfluidic paper-based analytical device
PAD: paper-based analytical device
PBS: phosphate buffer saline
POCT: *point-of-care* testing
px: pixel
Tf: transferrin
TIBC: total iron-binding capacity
TSAT: transferrin saturation percentage
UIBC: unsaturated iron-binding capacity

## References

[ref1] Elsayed M. E.; Sharif M. U.; Stack A. G. Transferrin Saturation: A Body Iron Biomarker. Adv. Clin. Chem. 2016, 75, 71–97. 10.1016/bs.acc.2016.03.002. 27346617

[ref2] Szoke D.; Panteghini M. Diagnostic Value of Transferrin. Clin. Chim. Acta 2012, 413, 1184–1189. 10.1016/j.cca.2012.04.021. 22546612

[ref3] Lopez A.; Cacoub P.; Macdougall I. C.; Peyrin-Biroulet L. Iron Deficiency Anaemia. Lancet 2016, 387, 907–916. 10.1016/S0140-6736(15)60865-0. 26314490

[ref4] Camaschella C. New Insights into Iron Deficiency and Iron Deficiency Anemia. Blood Rev. 2017, 31, 225–233. 10.1016/j.blre.2017.02.004. 28216263

[ref5] Castellanos M.; Puig N.; Carbonell T.; Castillo J.; Martinez J. M.; Rama R.; Dávalos A. Iron Intake Increases Infarct Volume after Permanent Middle Cerebral Artery Occlusion in Rats. Brain Res. 2002, 952, 1–6. 10.1016/S0006-8993(02)03179-7. 12363398

[ref6] García-Yébenes I.; Sobrado M.; Moraga A.; Zarruk J. G.; Romera V. G.; Pradillo J. M.; Perez De La Ossa N.; Moro M. A.; Dávalos A.; Lizasoain I. Iron Overload, Measured as Serum Ferritin, Increases Brain Damage Induced by Focal Ischemia and Early Reperfusion. Neurochem. Int. 2012, 61, 1364–1369. 10.1016/j.neuint.2012.09.014. 23036361

[ref7] Millán M.; Degregorio-Rocasolano N.; Pérez de la Ossa N.; Reverté S.; Costa J.; Giner P.; Silva Y.; Sobrino T.; Rodríguez-Yáñez M.; Nombela F.; Campos F.; Serena J.; Vivancos J.; Martí-Sistac O.; Cortés J.; Dávalos A.; Gasull T. Targeting Pro-Oxidant Iron with Deferoxamine as a Treatment for Ischemic Stroke: Safety and Optimal Dose Selection in a Randomized Clinical Trial. Antioxidants 2021, 10, 1270 10.3390/antiox10081270. 34439518 PMC8389327

[ref8] Yamanishi H.; Kimura S.; Iyama S.; Yamaguchi Y.; Yanagihara T. Fully Automated Measurement of Total Iron-Binding Capacity in Serum. Clin. Chem. 1997, 43, 2413–2417. 10.1093/clinchem/43.12.2413. 9439463

[ref9] Yamanishi H.; Iyama S.; Yamaguchi Y.; Kanakura Y.; Iwatani Y. Total Iron-Binding Capacity Calculated from Serum Transferrin Concentration or Serum Iron Concentration and Unsaturated Iron-Binding Capacity. Clin. Chem. 2003, 49, 175–178. 10.1373/49.1.175. 12507977

[ref10] Gambino R.; Desvarieux E.; Orth M.; Matan H.; Ackattupathil T.; Lijoi E.; Wimmer C.; Bower J.; Gunter E. The Relation between Chemically Measured Total Iron-Binding Capacity Concentrations and Immunologically Measured Transferrin Concentrations in Human Serum. Clin. Chem. 1997, 43, 2408–2412. 10.1093/clinchem/43.12.2408. 9439462

[ref11] Strzelak K.; Rybkowska N.; Wiśniewska A.; Koncki R. Photometric Flow Analysis System for Biomedical Investigations of Iron/Transferrin Speciation in Human Serum. Anal. Chim. Acta 2017, 995, 43–51. 10.1016/j.aca.2017.10.015. 29126480

[ref12] Frank C.; Rienitz O.; Jährling R.; Schiel D.; Zakel S. Reference Measurement Procedures for the Iron Saturation in Human Transferrin Based on IDMS and Raman Scattering. Metallomics 2012, 4, 1239–1244. 10.1039/c2mt20183f. 23151869

[ref13] Eleftheriadis T.; Liakopoulos V.; Antoniadi G.; Stefanidis I. Which Is the Best Way for Estimating Transferrin Saturation. Ren. Fail. 2010, 32, 1022–1023. 10.3109/0886022X.2010.502609. 20722575

[ref14] Scheiber-Mojdehkar B.; Lutzky B.; Schaufler R.; Sturm B.; Goldenberg H. Non-Transferrin-Bound Iron in the Serum of Hemodialysis Patients Who Receive Ferric Saccharate: No Correlation to Peroxide Generation. J. Am. Soc. Nephrol. 2004, 15, 1648–1655. 10.1097/01.ASN.0000130149.18412.56. 15153577

[ref15] Kitsati N.; Liakos D.; Ermeidi E.; Mantzaris M. D.; Vasakos S.; Kyratzopoulou E.; Eliadis P.; Andrikos E.; Kokkolou E.; Sferopoulos G.; Mamalaki A.; Siamopoulos K.; Galaris D. Rapid Elevation of Transferrin Saturation and Serum Hepcidin Concentration in Hemodialysis Patients after Intravenous Iron Infusion. Haematologica 2015, 100, e80–e83. 10.3324/haematol.2014.116806. 25425685 PMC4349282

[ref16] Angoro B.; Motshakeri M.; Hemmaway C.; Svirskis D.; Sharma M. Non-Transferrin Bound Iron. Clin. Chim. Acta 2022, 531, 157–167. 10.1016/j.cca.2022.04.004. 35398023

[ref17] Agarwal R. Transferrin Saturation with Intravenous Irons: An in Vitro Study. Kidney Int. 2004, 66, 1139–1144. 10.1111/j.1523-1755.2004.00864.x. 15327409

[ref18] DeGregorio-Rocasolano N.; Martí-Sistac O.; Ponce J.; Castelló-Ruiz M.; Millán M.; Guirao V.; García-Yébenes I.; Salom J. B.; Ramos-Cabrer P.; Alborch E.; Lizasoain I.; Castillo J.; Dávalos A.; Gasull T. Iron-Loaded Transferrin (Tf) Is Detrimental Whereas Iron-Free Tf Confers Protection against Brain Ischemia by Modifying Blood Tf Saturation and Subsequent Neuronal Damage. Redox Biol. 2018, 15, 143–158. 10.1016/j.redox.2017.11.026. 29248829 PMC5975212

[ref19] Drain P. K.; Hyle E. P.; Noubary F.; Freedberg K. A.; Wilson D.; Bishai W. R.; Rodriguez W.; Bassett I. V. Diagnostic Point-of-Care Tests in Resource-Limited Settings. Lancet Infect. Dis. 2014, 14, 239–249. 10.1016/S1473-3099(13)70250-0. 24332389 PMC4016042

[ref20] Karim K.; Lamaoui A.; Amine A. Paper-Based Optical Sensors Paired with Smartphones for Biomedical Analysis. J. Pharm. Biomed. Anal. 2023, 225, 115207 10.1016/j.jpba.2022.115207. 36584551

[ref21] Vashist S. K.; Luppa P. B.; Yeo L. Y.; Ozcan A.; Luong J. H. T. Emerging Technologies for Next-Generation Point-of-Care Testing. Trends Biotechnol. 2015, 33, 692–705. 10.1016/j.tibtech.2015.09.001. 26463722

[ref22] Martinez A. W.; Phillips S. T.; Butte M. J.; Whitesides G. M. Patterned Paper as a Platform for Inexpensive, Low-Volume, Portable Bioassays. Angew. Chem., Int. Ed. 2007, 46, 1318–1320. 10.1002/anie.200603817. PMC3804133 17211899

[ref23] Noviana E.; Ozer T.; Carrell C. S.; Link J. S.; McMahon C.; Jang I.; Henry C. S. Microfluidic Paper-Based Analytical Devices: From Design to Applications. Chem. Rev. 2021, 121, 11835–11885. 10.1021/acs.chemrev.0c01335. 34125526

[ref24] Yamada K.; Shibata H.; Suzuki K.; Citterio D. Toward Practical Application of Paper-Based Microfluidics for Medical Diagnostics: State-of-the-Art and Challenges. Lab Chip 2017, 17, 1206–1249. 10.1039/c6lc01577h. 28251200

[ref25] Shrivas K.; Monisha; Kant T.; Karbhal I.; Kurrey R.; Sahu B.; Sinha D.; Patra G. K.; Deb M. K.; Pervez S. Smartphone Coupled with Paper-Based Chemical Sensor for on-Site Determination of Iron(III) in Environmental and Biological Samples. Anal. Bioanal. Chem. 2020, 412, 1573–1583. 10.1007/s00216-019-02385-x. 31932862

[ref26] Serhan M.; Jackemeyer D.; Long M.; Sprowls M.; Diez Perez I.; Maret W.; Chen F.; Tao N.; Forzani E. Total Iron Measurement in Human Serum with a Novel Smartphone-Based Assay. IEEE J. Transl. Eng. Heal. Med. 2020, 8, 2800309 10.1109/JTEHM.2020.3005308. PMC7433848 32832281

[ref27] Dortez S.; Crevillen A. G.; Escarpa A. Integrated Calibration and Serum Iron in Situ Analysis into an Array Microfluidic Paper-Based Analytical Device with Smartphone Readout. Talanta 2023, 253, 123914 10.1016/j.talanta.2022.123914. 36103750

[ref28] Srinivasan B.; O’Dell D.; Finkelstein J. L.; Lee S.; Erickson D.; Mehta S. IronPhone: Mobile Device-Coupled Point-of-Care Diagnostics for Assessment of Iron Status by Quantification of Serum Ferritin. Biosens. Bioelectron. 2018, 99, 115–121. 10.1016/j.bios.2017.07.038. 28750335

[ref29] Carrilho E.; Martinez A. W.; Whitesides G. M. Understanding Wax Printing: A Simple Micropatterning Process for Paper-Based Microfluidics. Anal. Chem. 2009, 81, 7091–7095. 10.1021/ac901071p. 20337388

[ref30] Stookey L. L. Ferrozine–A new spectrophotometric reagent for iron. Anal. Chem. 1970, 42, 779–781. 10.1021/ac60289a016.

[ref31] Mori L.; Bekkering A.; Traini J.; Vanderlinden L. Ferrozine Iron and Total Iron-Binding Capacity Method Adapted to the ABA-100 Bichromatic Analyzer. Clin. Chem. 1981, 27, 1441–1444. 10.1093/clinchem/27.8.1441. 7273407

[ref32] Smith G. L.; Reutovich A. A.; Srivastava A. K.; Reichard R. E.; Welsh C. H.; Melman A.; Bou-Abdallah F. Complexation of Ferrous Ions by Ferrozine, 2,2 -Bipyridine and 1,10-Phenanthroline: Implication for the Quantification of Iron in Biological Systems. J. Inorg. Biochem. 2021, 220, 111460 10.1016/j.jinorgbio.2021.111460. 33866045

[ref33] Sierra T.; Crevillen A. G.; Escarpa A. Electrochemical sensor for the assessment of carbohydrate deficient transferrin: Application to diagnosis of congenital disorders of glycosilation. Biosens. Bioelectron. 2021, 179, 113098 10.1016/j.bios.2021.113098. 33636501

[ref34] Sierra T.; Henry C. S.; Crevillén A. G.; Escarpa A. Disposable Passive Electrochemical Microfluidic Device for Diagnosis of Congenital Disorders of Glycosylation. Anal. Sens. 2022, 2, e202100038 10.1002/anse.202100038.

[ref35] Breuer W.; Hershko C.; Cabantchik Z. I. The Importance of Non-Transferrin Bound Iron in Disorders of Iron Metabolism. Transfus. Sci. 2000, 23, 185–192. 10.1016/S0955-3886(00)00087-4. 11099894

