# Supplementary material for: Paper-Based Analytical Devices for Accurate Assessment of Transferrin Saturation in Diagnosed Clinical Samples from Ischemic Stroke Patients
Source: Anal Chem. 2023 Jul 24;95(33):12391–7. doi: 10.1021/acs.analchem.3c01982 (PMC10448438; doi:10.1021/acs.analchem.3c01982)
Supplement: Supplementary file 1 — ac3c01982_si_001.pdf [file ac3c01982_si_001.pdf]

Supporting Information

Paper-based analytical device for accurate assessment of transferrin saturation in diagnosed clinical samples from ischemic stroke patients

Silvia Dortez[a], Núria DeGregorio-Rocasolano[b], Mònica Millán[c], Teresa Gasull[b], Agustín G. Crevillen[d*], Alberto Escarpa[a,e*]

a. Department of Analytical Chemistry, Physical Chemistry and Chemical Engineering, University of Alcala, 28805 Alcala de Henares, Madrid, Spain.

b. Cellular and Molecular Neurobiology Research Group, Department of Neurosciences, Germans Trias I Pujol Research Institute (IGTP), 08916, Badalona, Barcelona, Spain.

c. Department of Neurociences, Germans Trias I Pujol University Hospital, Universitat Autònoma de Barcelona, 08916, Badalona, Barcelona, Spain.

d. Department of Analytical Sciences, Faculty of Sciences, Universidad Nacional de Educacion a Distancia (UNED), 28040, Madrid, Spain.

e. Chemical Research Institute "Andrés M. Del Río" (IQAR), University of Alcala, 28805, Alcala de Henares, Madrid, Spain.

Corresponding Authors

***Alberto Escarpa** – Department of Analytical Chemistry, Physical Chemistry and Chemical Engineering, University of Alcala, 28805, Alcala de Henares, Madrid, Spain; Chemical Research Institute "Andres M. Del Rio" (IQAR), University of Alcala, Madrid, 28805, Spain.
Email: alberto.escarpa@uah.es

***Agustín G. Crevillen** – Department of Analytical Sciences, Faculty of Sciences, Universidad Nacional de Educación a Distancia (UNED), 28040, Madrid, Spain.
Email: agustingcrevillen@ccia.uned.es

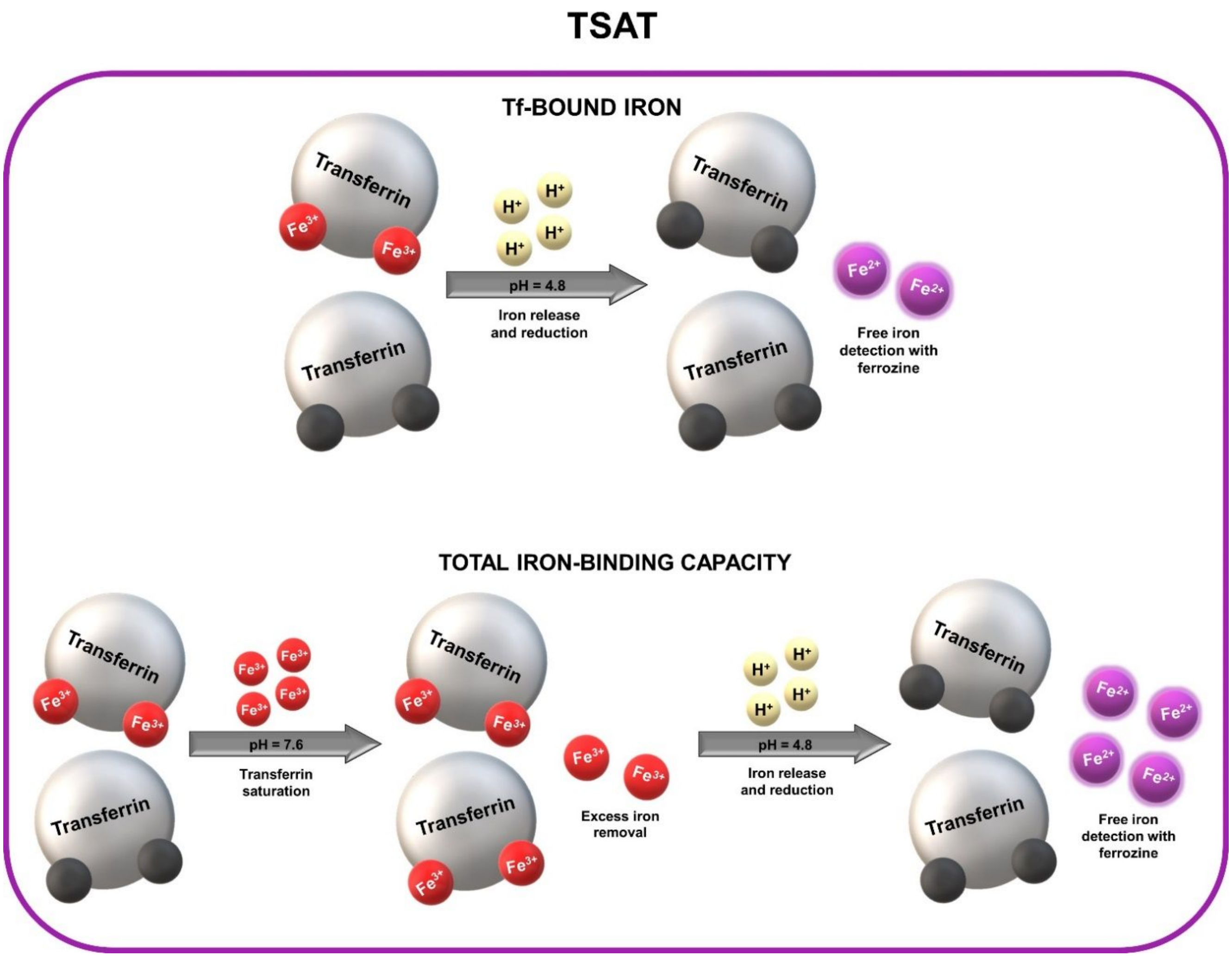

Figure S1. Scheme of the analytical reactions used for determination of TSAT.

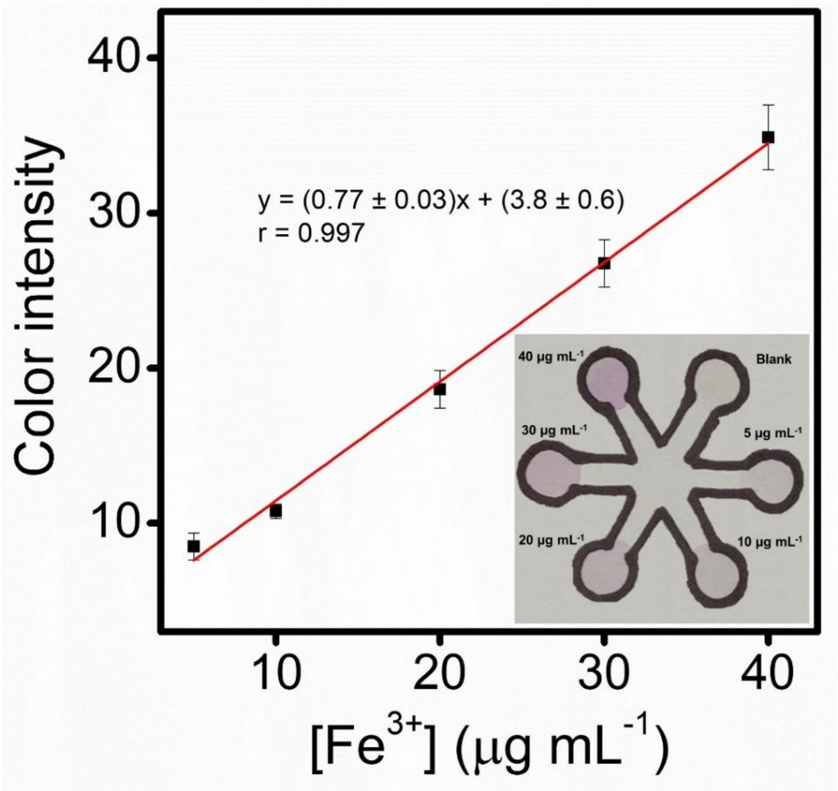

Figure S2. Calibration plots of color intensity *versus* the concentration of Fe^{3+} (n = 3). Inset: Image of a PAD used for calibration. Image of a PAD used for calibration curve of color intensity *versus* the concentration of Fe^{3+}. Experimental condition: see **Materials and Methods**.

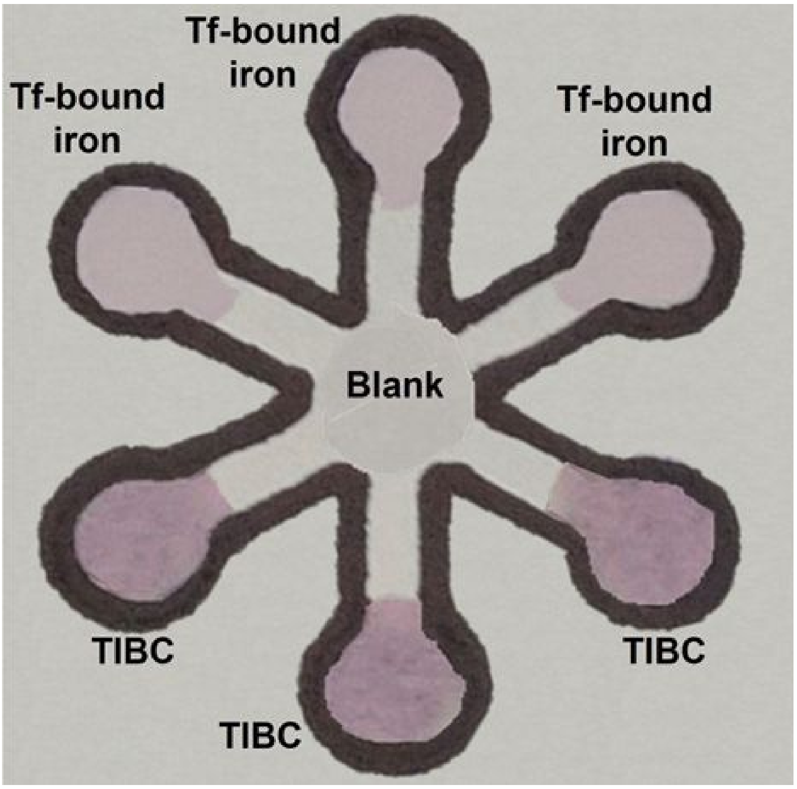

Figure S3. Validation of Tf-bound iron and TIBC by the analysis of a certified reference material (human serum) using the same PAD (n = 3). Experimental condition: see **Materials and Methods**.

Table S1. TSAT in serum samples from ischemic stroke patients obtained by PAD and by urea-PAGE (n = 3)

Sample	$TSAT_{PAD}$ (%)	$TSAT_{urea\text{-}PAGE}$ (%)[1]
S1	25 ± 1	18 ± 1
S2	15 ± 3	18 ± 4
S3	15 ± 1	21 ± 1
S4	28 ± 2	27 ± 6
S5	40 ± 6	29 ± 1
S6	29 ± 4	33 ± 7
S7	39 ± 2	34 ± 7
S8	35 ± 3	35 ± 3
S9	37 ± 3	35 ± 7
S10	36 ± 3	36 ± 6
S11	43 ± 1	38 ± 4
S12	37 ± 4	40 ± 3
S13	38 ± 4	40 ± 4
S14	40 ± 3	43 ± 4
S15	39 ± 5	43 ± 6
S16	40 ± 5	46 ± 1
S17	65 ± 7	61 ± 5
S18	61 ± 3	64 ± 6

▪ REFERENCES

(1) DeGregorio-Rocasolano, N.; Martí-Sistac, O.; Ponce, J.; Castelló-Ruiz, M.; Millán, M.; Guirao, V.; García-Yébenes, I.; Salom, J. B.; Ramos-Cabrer, P.; Alborch, E.; Lizasoain, I.; Castillo, J.; Dávalos, A.; Gasull, T. Iron-Loaded Transferrin (Tf) Is Detrimental Whereas Iron-Free Tf Confers Protection against Brain Ischemia by Modifying Blood Tf Saturation and Subsequent Neuronal Damage. *Redox Biol.* **2018**, *15*, 143–158. https://doi.org/10.1016/j.redox.2017.11.026.
